# Life-style characteristics and cardiovascular risk factors in regular downhill skiers: an observational study

**DOI:** 10.1186/1471-2458-13-788

**Published:** 2013-08-29

**Authors:** Martin Burtscher, Thomas Bodner, Johannes Burtscher, Gerhard Ruedl, Martin Kopp, Gregor Broessner

**Affiliations:** 1 Department of Sport Science, University of Innsbruck, Innsbruck, Austria; 2 Department of Neurology, Medical University of Innsbruck, Innsbruck, Austria; 3 Department of Pharmacology, Medical University of Innsbruck, Innsbruck, Austria; 4 Department of Sport Science, Medical Section, University of Innsbruck, Fürstenweg 185, A-6020 Innsbruck, Austria

**Keywords:** Downhill skiing, Elderly, Health effects, Cardiovascular risk factors, Memory complaints

## Abstract

**Background:**

Downhill skiing is part of active life style in many residents of Alpine regions. However, only very little information is available whether downhill skiing on a regular basis is associated with a healthier life style resulting in the reduction of major risk factors for cardiovascular diseases and memory deficits when compared to the general population. Thus, the aim of the study was to compare life-style characteristics and cardiovascular risk factors between regular downhill skiers and the general population.

**Methods:**

Self-reported health and life-style data were collected by questionnaire from 1259 long-term downhill skiers (971 males, aged 57.3 ± 14.6 years; 288 females, aged 47.7 ± 16.4 years) and compared with data from the general population.

**Results:**

Long-term skiers showed more favourable life-style characteristics and a better health status than the general population. Prevalences of hypercholesterolemia, systemic hypertension, diabetes, the frequency of mental stress and the occurrence of memory deficits declined with increasing yearly skiing frequency.

**Conclusion:**

Long-term alpine skiing on a regular basis may contribute to healthy aging by its association with a healthier life style.

## Background

Downhill skiing represents one of the most popular winter sports world-wide and particularly in Alpine regions. Annually, an estimated 8 million skiers visit the mountainous regions of Austria alone [1]. On the one hand, several studies have dealt with the risk of traumatic and non-traumatic events during downhill skiing [1-3]. On the other hand however, higher levels of leisure-time exercise are generally associated with a healthier life style [4] and thus downhill skiing as part of regular physical activity may contribute to well-being and longevity [5]. These beneficial consequences may at least partly be mediated by reducing the usually high prevalence of major cardiovascular risk factors in the general population [6]. In addition, the exposure to moderate altitude where skiing activities are typically performed might support these favourable implications [7]. However, most studies evaluating the effects of physical activity on the development of cardiovascular risk factors refer to aerobic type of exercises [8,9]. Downhill skiing however may differ from that because it is not primarily an aerobic exercise [10]. Data on potentially beneficial health impacts of downhill skiing are scarce [10] and to our knowledge no study has focussed on such impacts in the long term. The aim of the study was to compare life-style characteristics and cardiovascular risk factors between regular downhill skiers and the general population. We hypothesised that downhill skiing on a regular basis for many years may be associated with a healthier life style and the development of fewer traditional risk factors for cardiovascular diseases when compared to the general population. Because cardiovascular risk factors such as hypertension, dyslipidemia, and diabetes appear to increase the risk of cognitive impairment [11] we also expected a population of regular skiers to report less memory deficits.

## Methods

A health and life-style questionnaire (Additional file 1) has been designed including items selected from the Austrian microcensus survey program [12] from our previous study on the sudden cardiac death risk during alpine skiing [1,3] and from the Global Deterioration Scale [13]. The questionnaire has been tested in 15 male and female regular downhill skiers aged between 15 and 75 years and based on their comments it has been revised to optimize clarity and time-saving handling. Then it was distributed as a supplement in the Magazine of the Austrian Ski Federation. We particularly asked long-term downhill skiers for completing and returning the questionnaire. Data were collected on anthropometric characteristics, general well-being, life style factors like regular endurance exercises (hours/week), the annual frequency of downhill skiing (three categories for low, moderate and high skiing activity: < 11, 11–20, > 20 days/year), smoking (yes, no), alcohol intake (regularly, occasionally, no), self-rated unhealthy nutrition (yes, no) and known risk factors for cardiovascular diseases and selected symptoms of memory deficits. Risk factors for cardiovascular diseases included systemic hypertension (yes, no), hypercholesterolemia (yes, no), diabetes (yes, no), and frequent mental stress (yes, no). Memory deficits comprised forgetting familiar names, difficulty with learning new things, difficulty with concentrating, and difficulty with complex tasks [13]. These 4 symptoms had to be rated from 1 – 3 (no, occasionally or frequently). At least one symptom rated as “frequently” plus a total score greater than 6 was arbitrarily defined as memory deficits. Life-style and risk factors of the skier population were compared to those of an Austrian normal population of similar age and gender distribution recorded by the Austrian microcensus survey program (Austrian Health Interview Survey) [12]. The basis for the Austrian Health Interview Survey was the preliminary version of the European Health Interview Survey (EHIS), which is carried out every 5 years by the European Statistical System. The study has been approved by the institutional review board of the department of Sport Science of the University of Innsbruck.

### Statistics

Data are presented as means (SD) or frequencies and proportions. To examine risk factors, ANOVA or Student’s t-tests were used to compare means of continuous data and Chi-squared test for analysis of frequencies and proportions. The Cochran Armitage trend test was used to study the underlying trend. Using multivariate step-wise logistic regression analyses, anthropometric data, smoking, alcohol drinking and unhealthy nutrition habits, pain frequency, mental stress, aerobic exercise and the frequency of skiing were considered as potential predictors (independent variables) for the development of systemic hypertension, hypercholesterolemia, diabetes or memory complaints (binary dependent variables). Differences were considered statistically significant at P < 0.05.

## Results

The questionnaire has been returned by 1259 downhill skiers, 971 males and 288 females. As expected, mainly long-term skiers returned the questionnaire, which is confirmed by the impressive skiing history reported (Table 1). Thus, our sample represents a group of downhill skiers who performed their sport on a regular basis for many years.

**Table 1:** Health and life style characteristics of the study population

**Variable**	**Male skiers**	**Age matched**	**Female skiers**	**Age matched**
	**n = 971**	**General population**^**1)**^	**n = 288**	**General population**^**1)**^
Age (years)	57.3 (14.6; 16–90)	(30 – 75)	47.7 (16.4; 16 – 82)^b^	(30 – 60)
Weight (kg)	78.8 (10.7)		62.4 (9.3)^b^	
Height (cm)	176.4 (6.4)		165.6 (6.3)^b^	
Body mass index (> 25.0/< 25.1)	447/524 (46/54)	(56/44)^a^	47/241 (16/84)^b^	(45/55)^a^
Health state (moderate to bad/very good, good)	190/781 (20/80)	(30/70)^a^	34/254 (16/84)	(30/70)^a^
Smoker (yes/no)	97/874 (10/90)	(28/72)^a^	20/268 (7/93)	(19/81)^a^
Alcohol consumption (regularly/occasionally/no)	39/484/448 (4/50/46)	(38/34/28)^a^	6/84/198 (2/29/69)^b^	(15/35/50)^a^
Unhealthy nutrition (yes/no)	542/429 (56/44)		193/95 (67/33)^b^	
Hypercholesterolemia (yes/no)	347/624 (36/64)	(34/66)	76/212 (26/74)^b^	(28/72)
Systemic hypertension (yes/no)	255/716 (26/74)	(30/70)	31/257 (11/89)^b^	(14/86)
Diabetes (yes/no)	49/922 (5/95)	(6/94)	8/288 (3/97)	(4/96)
Pain frequency (regularly/occasionally/no)	47/835/89 (5/86/9)		14/248/26 (5/86/9)	
Frequent mental stress (yes/no)	566/405 (58/42)		104/184 (36/64)^b^	
Memory deficits (yes/no)	66/905 (7/93)		17/271 (6/94)	
Aerobic exercise (< 3/> 2 hours/week)	470/501 (48/52)	(72/28)^a^	146/142 (51/49)	(72/28)^a^
Skiing frequency (< 21/> 20 days/year)	341/630 (35/65)		126/162 (44/56)^b^	

Values are means (SD; range for age) and frequencies (proportions).

^1)^Data are available from the Austrian microcensus survey program 2006/7; Ref. [12].

^a^Proportions are different (P < 0.05; Chi-squared test) between skiers and the age matched general population.

^b^Means or proportions are different (P < 0.05; t-test or Chi-squared test) between male and female skiers.

### Health and life-style characteristics of the study population

Anthropometric data, health and life style characteristics are presented in Table 1. Male skiers reported an average skiing history of 48.3 years and female skiers of 37.8 years (P < 0.05); males being older, heavier, taller and with a higher BMI than females (all P < 0.05). Compared to female skiers, males reported to more often drink alcohol, reported more frequently unhealthy nutrition, showed a higher prevalence of known systemic hypertension, hypercholesterolemia and mental stress and had more skiing days per year (all P < 0.05).

### Comparison of health and life-style characteristics between long-term skiers and the general population

In comparison to a general population of similar age, both male and female skiers reported a better health status, drank alcohol less frequently, were more often non-smokers and performed aerobic exercise more frequently than the general population. However, the prevalence of reported risk factors for cardiovascular diseases did not differ between the general population and the skiers (Table 1).

### Health and life-style characteristics of the study population related to the yearly skiing frequency

The general health status improved with increasing yearly skiing frequency, the prevalence of self estimated unhealthy nutrition habits, known hypercholesterolemia, systemic hypertension, diabetes, the frequency of mental stress and the occurrence of memory deficits decreased with increasing skiing frequency (all P for trend < 0.05; Table 2). The weekly amount of aerobic exercise increased with higher skiing frequency (P for trend < 0.05).

**Table 2:** Health and life style characteristics of the study population related to the yearly skiing frequency

**Variable**	**Skiing frequency (days/year)**			**P-Value**
	**1-10**	**11-20**	**> 20**	
	**n = 179**	**n = 293**	**n = 787**	
Age; years (mean; SD)	55.0; 14.9	55.2; 15.0	55.1; 15.3	0.92
Body mass index (> 25.0; %)	76; 42	119; 41	299; 38	0.47
Health state; moderate or bad (n; %)	51; 28	61; 21	112; 14	< 0.001 ^a^
Smoker (n; %)	21; 12	26; 9	70; 9	0.48
Alcohol intake; regularly or occasionally (n; %)	94; 53	155; 53	364; 46	0.08
Unhealthy nutrition (n; %)	89; 50	122; 42	293; 37	< 0.01 ^a^
Hypercholesterolemia (n; %)	77; 43	110; 38	236; 30	< 0.01 ^a^
Systemic hypertension (n; %)	54; 30	70; 24	162; 21	< 0.01 ^a^
Diabetes (n; %)	15; 8	16; 5	26; 3	< 0.01 ^a^
Pain frequency; regularly or occasionally (n; %)	163; 91	262; 89	719; 91	0.61
Frequent mental stress (n; %)	106; 59	168; 57	396; 50	0.03 ^a^
Memory deficits (n; %)	21; 12	24; 8	38; 5	< 0.01 ^a^
Aerobic exercise (< 3 hours/week) (n; %)	104; 58	179; 58	342; 43	< 0.001 ^a^

Data are means (age) or frequencies and proportions (all other variables). P-values have been calculated by ANOVA (age) and Chi-squared test for analysis of frequencies.

^a^Means P < 0.05 for trend (Cochran Armitage trend test).

### Independent predictors for the occurrence of various risk factors

The results of the multivariate step-wise logistic regression analyses, adjusted for age and gender, are presented in Table 3. Alcohol intake increased (OR: 1.59; 95% CI: 1.25 – 2.01) and the higher yearly skiing frequency decreased (OR: 0.56; 0.39 – 0.80) the chance for development of hypercholesterolemia in the studied skier population. The chance for the development of systemic hypertension was increased by a BMI > 25 (OR: 1.89; 1.40 – 2.55), frequent mental stress (OR: 1.39; 1.10 – 1.93), and was decreased by frequently performing aerobic exercise (OR: 0.59; 0.35 – 0.95). The chance for the development of diabetes type 2 was increased by a BMI > 25 (OR: 2.88; 1.60 – 5.20), and decreased by a high frequency of yearly skiing (OR: 0.74; 0.55 – 0.94). The chance for the occurrence of memory complaints was elevated by frequent suffering from pain (OR: 1.73 (1.28 – 2.33) and by hypercholesterolemia (OR: 1.75 (1.16 – 2.31), and was diminished by the increasing frequency of yearly skiing (11–20 days, OR: 0.37 (0.08 – 0.88); > 20 days: OR: 0.22 (0.09 – 0.64).

**Table 3:** Results of the multivariate logistic regression analyses

**Dependent variable**	**Predictors**	**Odds ratio (95% CI)**
Memory complaints	Pain frequency (regularly or occasionally versus none)	1.73 (1.28 – 2.33)
	Hypercholesterolemia (yes versus no)	1.75 (1.59 – 2.31)
	Skiing frequency (10–20 days versus < 10 days/year)	0.37 (0.08 – 0.88)
	Skiing frequency (> 21 days versus < 10 days/year)	0.22 (0.09 – 0.64)
Hypercholesterolemia	Alcohol intake (regularly or occasionally versus none)	1.59 (1.25 – 2.01)
	Skiing frequency (> 21 days versus < 10 days/year)	0.56 (0.39 – 0.80)
Systemic hypertension	BMI (> 25 versus < 25.1)	1.89 (1.40 – 2.55)
	Frequent mental stress (yes versus no)	1.39 (1.10 – 1.93)
	Aerobic exercise (> 3 hours versus < 3.1 hours/week)	0.59 (0.35 – 0.95)
Diabetes type 2	BMI (> 25.0 versus < 25.1)	2.88 (1.60 – 5.20)
	Skiing frequency (> 21 days versus < 10 days/year)	0.74 (0.55 – 0.94)

## Discussion

The findings of the present study demonstrate (1) a healthier life style of regular alpine skiers compared to the overall population, (2) an improved health status with increasing annual skiing frequency, and (3) the predictive importance of the yearly skiing frequency for the development of hypercholesterolemia, diabetes type 2, and memory complaints.

### Regular downhill skiing, life-style characteristics and cardiovascular risk factors

Although the studied skier population reported on average a healthier life-style (greater amount of aerobic exercise, less smoking, and less alcohol consumption) in comparison to the general population, the prevalence of cardiovascular risk factors (hypercholesterolemia, systemic hypertension, and diabetes) did not differ between the two populations. Thus, the healthier life style of downhill skiers did not seem to translate proportionally to reduced prevalence of cardiovascular risk factors. However, the prevalence of these risk factors decreased with increasing annual skiing frequency. Thus, our investigation shows a significant “dose dependent” effect of downhill skiing on self-reported cardiovascular risk factors and memory deficits. Differences observed between male and female skiers are similar to the gender differences demonstrated for the general population and may in the present study at least partly be explained by age effects. Of course, not only downhill skiing activities may have contributed to the demonstrated beneficial effects but rather the generally healthier life style associated with long-term regular downhill skiing. Nevertheless, the increasing yearly skiing frequency was an independent predictor for the reduced prevalence of hypercholesterolemia, diabetes type 2, and memory complaints.

It has been long known that higher levels of leisure-time physical activity are generally associated with a healthier life-style [4,14]. Several studies reported a threshold dose, e.g. of physical activity, to elicit beneficial effects on the lipid profile, blood pressure and blood glucose levels or aerobic exercise capacity [15-18] and skiing activities may have contributed to reach this threshold dose [19]. We recently demonstrated that more than 2 exercise sessions per week are necessary to elicit an improvement of fitness and related beneficial effects [15]. Thus, especially in the winter season skiing activities may well help to reach this threshold. The specific effectiveness of downhill skiing on the development of risk factors is supported by the evidence that eccentric type of exercise may have beneficial effects on lipid concentrations and glucose tolerance [18]. These findings would help to explain the predictive importance of skiing on the prevalence of hypercholesterolemia and diabetes and probably on the prevalence of cognitive complaints. Additionally, also short-term exposures to altitude may add to beneficial health effects by improving glycemic control, blood lipid profile or exercise tolerance [7,20]. The reduced prevalence of memory complaints reported by regular downhill skiers can at least partly explained by the current evidence indicating an association between cardiovascular risk factors and cognitive decline [11,21]. Besides, repeated exposures to hypoxia result in preconditioning likely associated with neuroprotection and neurogenesis [22]. Last but not least, social, emotional and intellectual benefits from outdoor recreation may help promote a healthy lifestyle.

### Limitations

We are unable to estimate the response rate of the overall population of long-term downhill skiers who really received and noticed the questionnaire. However, the large number of analyzed questionnaires strengthens the presented study findings but we cannot entirely exclude a potential systematic self-report bias. Furthermore, keeping our questionnaire as comprehensive as possible, we did not include questions about socioeconomic status and therefore cannot exclude that people who are skiers are on a higher socioeconomic level what might be relevant for health issues. Also the data on risk factors are self-reported data and we cannot rule out a certain number of unreported cases. However, data collection was very similar in the skier population and the overall population as well. Based on our practical experience we assume that the relatively low proportion of responding females may actually mirror the gender ratio of long-term skiers. Additionally, the presented findings only apply to regular downhill skiers who have performed their sport for many years but not to those who intend to start downhill skiing at an advanced age.

## Conclusion

Long-term downhill skiing on a regular basis is associated with a healthier life style in comparison to the general population. The general health status of long-term downhill skiers improves with increasing yearly skiing frequency by reducing certain cardiovascular risk factors and memory deficits.

## Consent

Written informed consent was obtained from the patient’s guardian/parent/next of kin for the publication of this report and any accompanying images.

## Competing interests

The authors declare that they have no competing interests.

## Authors’ contributions

MB conceptualized the study, performed statistical analyses and drafted the manuscript. TB contributed to the interpretation of the results and discussion. JB collected research data and was responsible for data entry and data control. GR contributed to the interpretation of the results and was responsible for critical reading of the final manuscript. MK and GB contributed to data analyses, the interpretation of the results and discussion. All authors have read and approved the final manuscript.

## Pre-publication history

The pre-publication history for this paper can be accessed here:

http://www.biomedcentral.com/1471-2458/13/788/prepub

## Supplementary Material

Additional file 1 Health and life-style questionnaire. Click here for file
