# Supplementary material for: Life-style characteristics and cardiovascular risk factors in regular downhill skiers: an observational study
Source: BMC Public Health. 2013 Aug 29;13:788. doi: 10.1186/1471-2458-13-788 (PMC3765782; doi:10.1186/1471-2458-13-788)
Supplement: Additional file 1 — Health and life-style questionnaire. [file 1471-2458-13-788-S1.pdf]

Anhang

Fragebogen zu Lebensstil und Gesundheitszustand von Alpinskifahrern

Bitte machen Sie ein Kreuz in das zutreffende Kästchen oder tragen Sie die Zahl ein. Danke!

Alter: ……… Jahre
Geschlecht: m ☐ w ☐
Größe: ………. cm
Gewicht: …..…. kg

Wie würden Sie Ihren Gesundheitszustand beschreiben?

1☐	2 ☐	3 ☐	4 ☐	5 ☐
Sehr gut	gut	zufriedenstellend	weniger gut	schlecht

An wie vielen Tagen waren Sie in den letzten 12 Monaten so krank, dass Sie das Bett hüten mussten?

An keinem Tag ☐ Oder: an ca. ☐ Tagen

Welche der folgenden Risikofaktoren liegen bei Ihnen vor?

	nicht	mäßig	stark
Rauchen	☐	☐	☐
Übergewicht	☐	☐	☐
Bewegungsmangel	☐	☐	☐
Falsche Ernährung	☐	☐	☐
Stress und Hektik	☐	☐	☐
Alkoholkonsum	☐	☐	☐
	☐	☐	☐
Hoher Blutdruck	☐	☐	☐
Hohes Cholesterin	☐	☐	☐
Erhöhter Blutzucker (Diabetes)	☐	☐	☐

Wie oft leiden Sie an Schmerzen?

1☐	2 ☐	3 ☐	4 ☐	5 ☐
Nie	selten	immer wieder	häufig	sehr häufig

Die Schmerzen betreffen:

Kopf	☐	Schultergelenk	☐
Zähne	☐	Ellbogengelenk	☐
Hals	☐	Handgelenk	☐
Brust	☐	Hüfte	☐
Bauch	☐	Kniegelenk	☐
Wirbelsäule	☐	Sprunggelenk	☐

Sonstige: ☐

Sie haben Schmerzen:

Beim Treppensteigen ☐
Bei längerem Gehen ☐
Beim Aufstehen nach längerem Sitzen ☐
Wenn Sie schwer Heben oder Tragen ☐

Sonsige: ☐

Trifft zu, dass Sie:	nie	gelegentlich	häufig
Bekannte Personen und Namen vergessen	☐	☐	☐
Sich schwer konzentrieren können	☐	☐	☐
Komplizierte Dinge langsam begreifen	☐	☐	☐
Sich neue Dinge nicht gut einprägen können	☐	☐	☐

Wie beurteilen Sie Ihr Skifahrkönnen?

1 ☐ Sehr gut 2 ☐ gut 3 ☐ zufriedenstellend 4 ☐ weniger gut 5 ☐ schlecht

Seit wie vielen Jahren fahren Sie Ski?

☐ Jahre

Wie oft haben Sie sich schon beim Skifahren verletzt, dass Sie einen Arzt aufsuchen mussten?

☐ Nie ☐ 1 mal ☐ 2-3 mal ☐ 4-5 mal ☐ 5-10 mal ☐ mehr als 10 mal

Wie oft fahren Sie pro Jahr Ski?

☐ 1-3 Tage ☐ 4-10 Tage ☐ 10-20 Tage ☐ mehr als 20 Tage

Wie viele Stunden pro Woche machen Sie Ausdauersport (Wandern, Joggen, Skilanglauf, Schwimmen, etc.)

☐ Weniger als 1 Stunde ☐ 1-2 Stunden ☐ 2-3 Stunden ☐ 4-5 Stunden ☐ mehr als 5 Stunden
